# Finite element modeling and in vivo analysis of electrode configurations for selective stimulation of pudendal afferent fibers

**DOI:** 10.1186/1471-2490-10-11

**Published:** 2010-05-25

**Authors:** John P Woock, Paul B Yoo, Warren M Grill

**Affiliations:** 1 Department of Biomedical Engineering, Duke University, Box 90281, Durham, NC 27708-0281, USA

## Abstract

**Background:**

Intraurethral electrical stimulation (IES) of pudendal afferent nerve fibers can evoke both excitatory and inhibitory bladder reflexes in cats. These pudendovesical reflexes are a potential substrate for restoring bladder function in persons with spinal cord injury or other neurological disorders. However, the complex distribution of pudendal afferent fibers along the lower urinary tract presents a challenge when trying to determine the optimal geometry and position of IES electrodes for evoking these reflexes. This study aimed to determine the optimal intraurethral electrode configuration(s) and locations for selectively activating targeted pudendal afferents to aid future preclinical and clinical investigations.

**Methods:**

A finite element model (FEM) of the male cat urethra and surrounding structures was generated to simulate IES with a variety of electrode configurations and locations. The activating functions (AFs) along pudendal afferent branches innervating the cat urethra were determined. Additionally, the thresholds for activation of pudendal afferent branches were measured in α-chloralose anesthetized cats.

**Results:**

Maximum AFs evoked by intraurethral stimulation in the FEM and in vivo threshold intensities were dependent on stimulation location and electrode configuration.

**Conclusions:**

A ring electrode configuration is ideal for IES. Stimulation near the urethral meatus or prostate can activate the pudendal afferent fibers at the lowest intensities, and allowed selective activation of the dorsal penile nerve or cranial sensory nerve, respectively. Electrode location was a more important factor than electrode configuration for determining stimulation threshold intensity and nerve selectivity.

## Background

Pudendal nerve stimulation is a potential means of restoring bladder function to persons with spinal cord injury (SCI). Stimulation of sensory (afferent) fibers either in the dorsal penile branch (DNP) or the cranial sensory branch (CSN) of the pudendal nerve can evoke stimulation frequency-dependent contraction or relaxation of the urinary bladder in cats [1,2]. However, the existence of comparable reflexes in persons with SCI remains unclear. In both experimental and clinical settings, intraurethral electrical stimulation (IES) has been utilized as a minimally invasive method to investigate these reflexes. However, the activation of multiple nerve pathways (pudendal and pelvic) by this approach did not enable identification of the specific sensory nerves responsible for the evoked bladder reflexes. The present study used a finite element model (FEM) and parallel *in vivo *measurements in the male cat to quantify the effects of electrode configuration and position on intraurethral activation of pudendal afferent nerve fibers. The primary aim of this study was to determine the optimal IES electrode configuration and stimulation locations for selectively activating pudendal afferents to aid future preclinical and clinical investigations.

Clinical evaluation of the bladder response to pudendal nerve stimulation is difficult because of the limited access to the pudendal nerve. The pudendal nerve trunk is located in the ischiorectal fossa, where it exhibits a complex and highly variable branching pattern that provides the motor and sensory innervation of the genitalia, urethra, rectum and the pelvic floor [3-8]. As a result of the complex nerve anatomy, clinical investigation of specific pudendal afferent fibers has been difficult. Transcutaneous stimulation (with external surface electrodes) of the DNP in humans can evoke robust bladder relaxation and promote continence [9-11], but this approach is limited to activation of superficial pudendal afferent branches. Percutaneous stimulation can activate the pudendal nerve [12,13] in humans, but it is unclear which branches of the nerve are activated. In contrast, surgically implanted cuff electrodes enable selective activation of the different pudendal nerve branches in the cat [2]. IES in the proximal urethra can evoke bladder contraction in humans [14], but the conflicting results between the human and cat [15,16] suggests that further analysis of the effects of intraurethral stimulation is necessary.

The goal of this study was to develop a computer model of IES that can be used to interpret data and guide design of IES electrode geometries for selective stimulation of pudendal afferents. We developed three-dimensional (3-D) FEMs to determine the electric potentials generated along the DNP and CSN by IES. The potentials were used to calculate the second spatial derivative of the extracellular potential along the nerve fibers (the 'activating function', AF [17]). The model and *in vivo *stimulation thresholds provide insight into the effects of electrode geometry and location valuable for future clinical and preclinical experiments investigating the ability to restore control of bladder function in persons with spinal cord injuries or other neurological disorders via stimulation of pudendal afferents.

## Methods

### Finite element modeling

**Figure 1:**
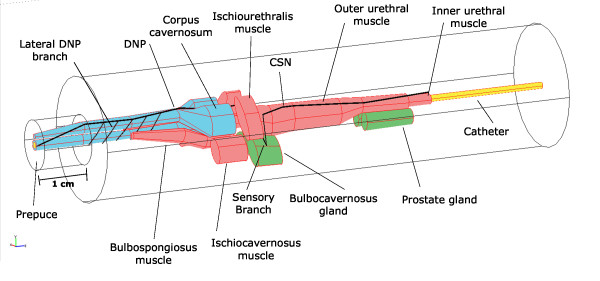
**Three dimensional model of the male cat urethra and surrounding structures**. The sensory branch of the pudendal nerve splits into proximal (cranial sensory nerve, CSN) and penile (dorsal nerve of the penis, DNP) branches. The nerve locations shown were used for calculating the activating function and were not physically included in the finite element model. The model was encased in a cylinder and bounding box (not shown) to allow for finer element sizes around the model components and larger elements near the outer boundaries. Scale = 1 cm at prepuce.

**Figure 2:**
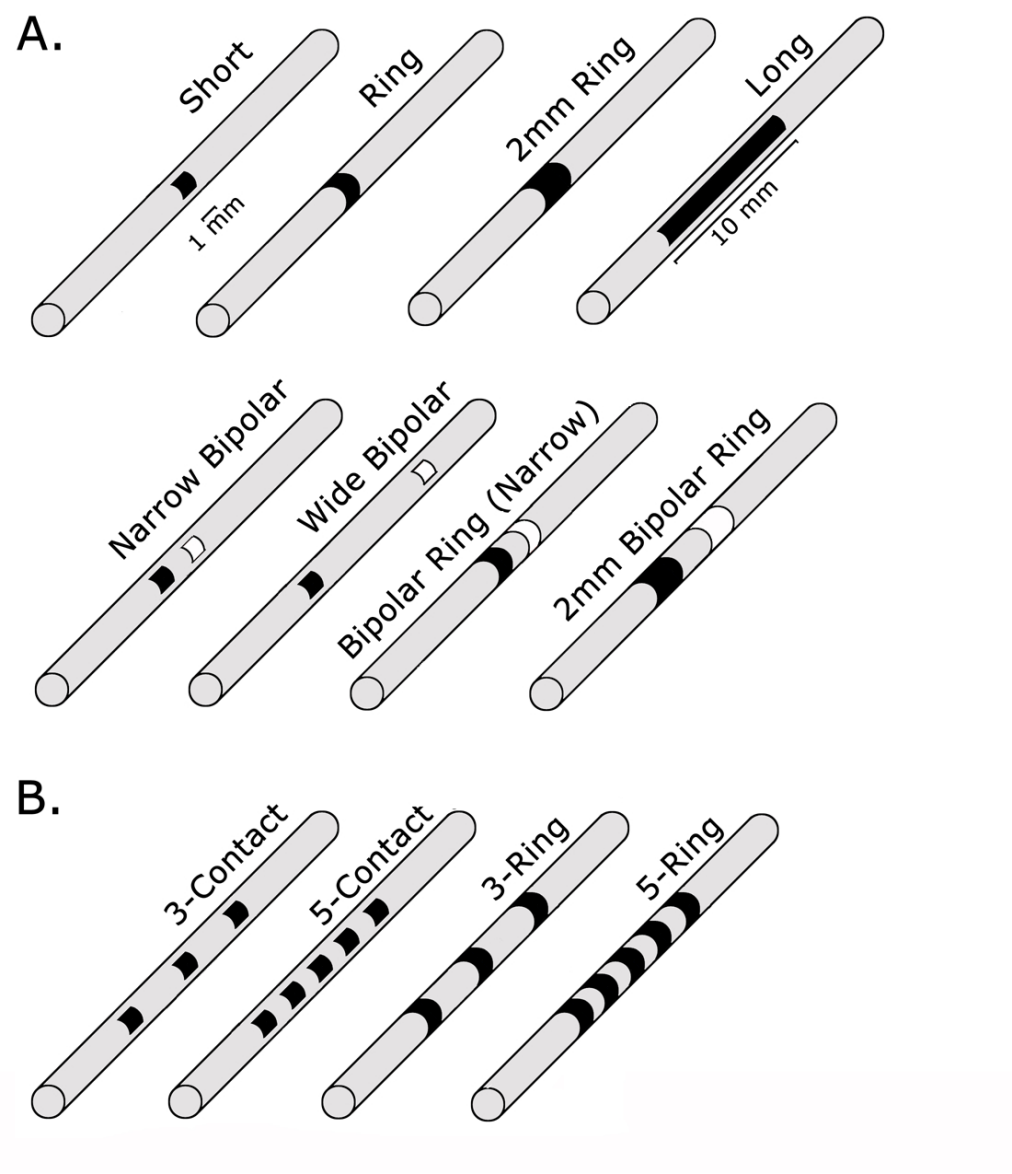
**Intraurethral catheter electrode configurations**. (A) Intraurethral electrical stimulation was simulated at 1-7 cm from the urethral meatus with eight different electrode configurations. (B) Four additional electrode configurations were simulated only at 2 cm from the urethral meatus for inclusion in the DNP branch analysis. In vivo stimulation was done with 1 mm ring, 2 mm ring, 3-ring, and 2 mm bipolar ring electrodes.

A 3-D model of the male cat urethra and surrounding structures was developed. The model spanned from the urethral meatus to 0.5 cm proximal to the prostate (Figure 1). Surrounding structures included the prepuce, corpus spongiosum, corpus cavernosum, bulbospongiosus muscle, bulbocavernosus glands, ischiocavernosus muscle, inner urethral muscle, outer urethral muscle, and prostate glands. The dimensions of the structures were determined from urethral cross-sections and gross anatomical observations [18,19]. The electrical properties of the tissues were taken from the literature (Table 1) and, for simplification, were all modeled as isotropic. The model included a 3.5 Fr intraurethral catheter with different electrode configurations (summarized in Figure 2) and was enclosed within a conducting medium.

**Table 1:** Electrical properties of the finite element model of the cat urethra

*Tissue type*	*Structures*	*Conductivity*	*Source*
	Urethral catheter	0.1 nS m^-1^	[29,30]

**Muscle tissue**	Bulbospongiosus m., ischiocavernosus m., ischiourethralis m., inner urethral m., outer urethral m.	0.291 S m^-1^	[31]

**Erectile tissue**	Corpus cavernosum, corpus spongiosum	0.6 S m^-1^	[31,32]

**Glands**	Bulbocavernosus g., prostate g.	0.4 S m^-1^	[31,32]

**Connective tissue, etc.**	Prepuce, bounding box	0.05 S m^-1^	[33]

The model was implemented in COMSOL Multiphysics (version 3.4) and partitioned into mesh elements using the finite element method. The internal tissue boundaries were set so that continuity of current was preserved, and the external boundaries were set to ground (V = 0) with the exception of the external boundaries of the prepuce and the adjacent wall of the bounding box, which were set to be electrically insulated (current density = 0). Increasing the mesh density around the electrode or doubling the bounding box size had minimal effect on the maximum potentials generated at the nerves (<5% change). The electrical input was a 1 mA cathodic regulated current for all simulations, and the model was solved using the conjugate gradient method.

### Nerve Modeling

The anatomical courses of the DNP and CSN branches of the pudendal nerve were modeled in Matlab (R2007a, Mathworks) based on previous anatomical data [18-21]. The nerves were represented bilaterally as single trunks, and lateral branches of the DNP were later included for further examination of DNP activation in the penile urethra. The potentials generated by IES were exported from the FEM model to Matlab and the potentials along the nerve paths were determined at 0.1 mm increments using interpolation.

### The Activating Function

The second spatial derivative of the extracellular potentials, *V*_*e*_, or the activating function (AF), was calculated along the modeled paths of the DNP and CSN to estimate neural activation. (Rattay 1989).

where *n *is the node of interest and ℓ = 0.5 *mm *is the internodal length assuming the modeled nerve fibers were 5 μm in diameter [11]. At the termination of the nerve fibers, the activating function was the first spatial derivative of the extracellular potential. The maximum AF was determined for the DNP and CSN bilaterally for each of 5 possible locations of the first (most distal) node in the fiber, and the resulting maximum AFs were averaged.

For analysis of activation of the lateral branches of the DNP in the penile urethra, the AFs were calculated along 5 lateral DNP branches (Figure 1) in addition to the DNP trunk. The branches were initially spaced 3 mm apart [19] and were located ~1.4 - 2.6 cm from the urethral meatus. The maximum AFs were calculated for each branch for all electrode configurations (Figure 2), and this was repeated for 50 sets of random branch locations generated by randomly varying the location of each branch within ± 1 cm along the longitudinal axis of the urethra. Maximum branch AFs were ordered from greatest to smallest for each of the 50 trials. The AFs were averaged across trials based on their rank (i.e., the maximum AF across the 5 branches was averaged over the 50 trials and so on for the 2^nd ^largest, etc).

### Selectivity Analysis

The effects of intraurethral electrode configurations on the ability to activate nerve fibers selectively was quantified by computing both the ratio of the AFs (AFR) under different conditions and the "selectivity", defined as the quotient of a minimum estimate of the maximum AF (mean, *μ*_*AF*,1_, minus one standard deviation, *σ*_*AF*,1_) generated by one set of stimulation conditions and a maximum estimate of the maximum AF (mean, *μ*_*AF*,2_, plus one standard deviation, *σ*_*AF*,2_) generated by a second set of stimulation conditions,

An AFR >1.5 combined with a selectivity >1 suggested, conservatively, that activation of a population of fibers under the first set of conditions could be achieved at a lower threshold than under the second set of conditions. If the AFR was <1.5 or selectivity was <1, it is likely that variation in nerve location, anatomical dimensions, and other factors would render stimulation thresholds under the two conditions indistinguishable during *in vivo *IES.

### In vivo experiments

Animal care and experimental procedures were approved by the Duke University Institutional Animal Care and Use Committee. Experiments were performed on 13 sexually intact adult male cats (2.8-4.6 kg) anesthetized with ketamine KCl (35 mg/kg i.m.) and α-chloralose (65 mg/kg i.v. supplemented at 15 mg/kg as needed). Artificial respiration maintained the end tidal CO_2 _between 3.5 and 4.0%, IV fluids (lactated Ringer's solution or saline/5% dextrose/sodium bicarbonate solution) were delivered at 15 cc/kg/hr via a catheter in the cephalic vein, and a thermostatic heating pad was used to maintain body temperature at ~38°C. Blood pressure was monitored through a catheter in the carotid artery. A catheter was inserted into the bladder dome and the bladder was drained externally to maintain an empty bladder.

A 3.5 or 5 Fr catheter modified with platinum electrodes embedded at 2 cm from the tip was inserted into the urethral meatus. The 3.5 Fr electrode included three 1 mm rings spaced 3.5 mm apart. The 5 Fr electrode included two 2 mm rings spaced 2 mm apart. Electrical stimulation (1 Hz) was applied with the catheter electrodes located 1-7 cm from the urethral meatus. Stimulation intensity varied from 0.5-15 mA, and the intensity threshold to evoke a reflex electromyographic response in the external anal sphincter (EAS EMG) was measured in 0.5 mA increments.

## Results

### Finite element model of intraurethral stimulation

Intraurethral stimulation was applied to electrodes positioned 1-7 cm from the urethral meatus using the electrode configurations shown in Figure 2A. The spatial distribution of the electric potential varied depending on the electrode configuration. Stimulation with the short electrode generated the largest voltage gradient along the urethra, both in maximum value and the volume of tissue that experienced a >1V change in potential, while stimulation with the 1 mm bipolar ring electrode generated the smallest change in potential. The orientation of the short and long electrodes resulted in greater potential changes in the dorsal direction (towards the nerves), whereas the potential changes generated by the ring electrode were more balanced across the dorsal and ventral directions.

### Activation threshold depended on electrode location

**Figure 3:**
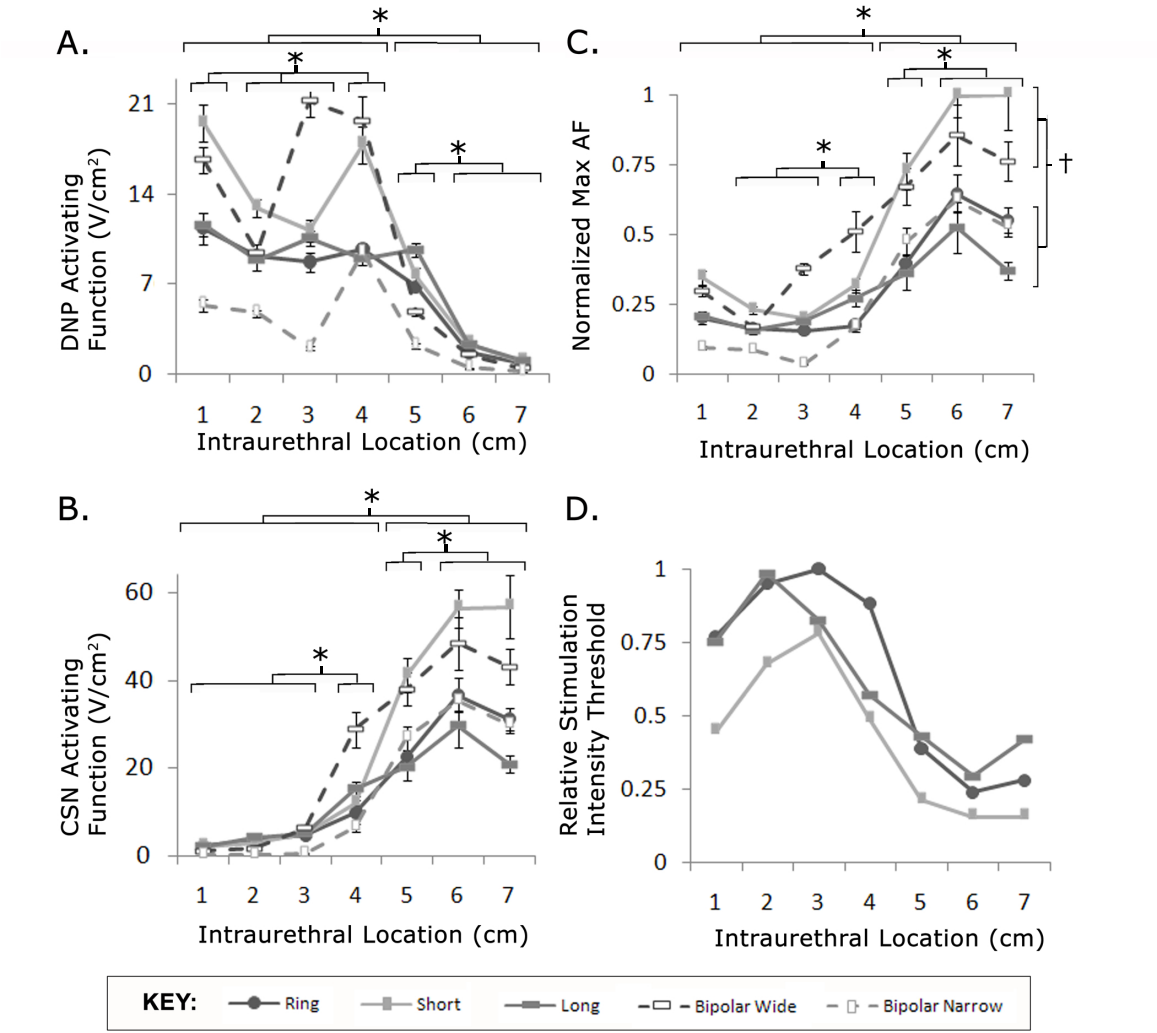
**Maximum activating functions (AFs) evoked along the DNP and CSN by intraurethral stimulation**. (A) The maximum AFs evoked along the DNP for intraurethral stimulation 1-7 cm from the urethral meatus. (B) Maximum AFs evoked along the CSN. (C) Maximum AFs evoked at each intraurethral stimulation location for combined DNP and CSN AFs, normalized by dividing by maximum AF over all locations and electrode configurations. (D) Relative stimulation threshold for short, ring, and long electrode configurations. Relative thresholds were determined from the relative values of the inverse of the maximum AFs. (A-C) Maximum AFs were dependent on stimulation location and electrode for the DNP AFs, CSN AFs, and combined DNP and CSN AFs (two-way ANOVA for each, p < 0.0001). (*) indicates significant difference between AFs evoked at different locations (p < 0.05, post-hoc comparison with Bonferroni correction). (C) (†) indicates significant different between AFs evoked with different electrode configurations (p < 0.05, Bonferroni post-hoc comparison).

The maximum value of the AF generated along the DNP and CSN varied depending on the location of stimulation (two-way ANOVA, p < 0.0001 for both nerves). The DNP AFs (Figure 3A) were significantly greater in the penile urethra (1-4 cm) than in the membranous urethra (5-7 cm), while stimulation in the membranous urethra (5-7 cm) generated significantly greater CSN AFs (Figure 3B) than stimulation in the penile urethra (1-4 cm). These results indicate that the DNP is more readily activated by stimulation in the penile urethra while the CSN is more readily activated by stimulation in the membranous urethra, which reflects the proximity of each nerve trunk to the corresponding segment of the urethra.

The maximum AFs, regardless of whether they occurred at the DNP or CSN, also depended on the electrode location (Figure 3C, two-way ANOVA, p < 0.0001). The maximum AFs for stimulation in the membranous urethra (5-7 cm) were significantly larger than those for stimulation in the penile urethra (1-4 cm). Relative stimulation thresholds were determined by inverting the AFs, and the simulation results suggest that the CSN is activated at lower thresholds by stimulation in the membranous urethra than the thresholds necessary to activate the DNP by stimulation in the penile urethra (Figure 3D).

**Figure 4:**
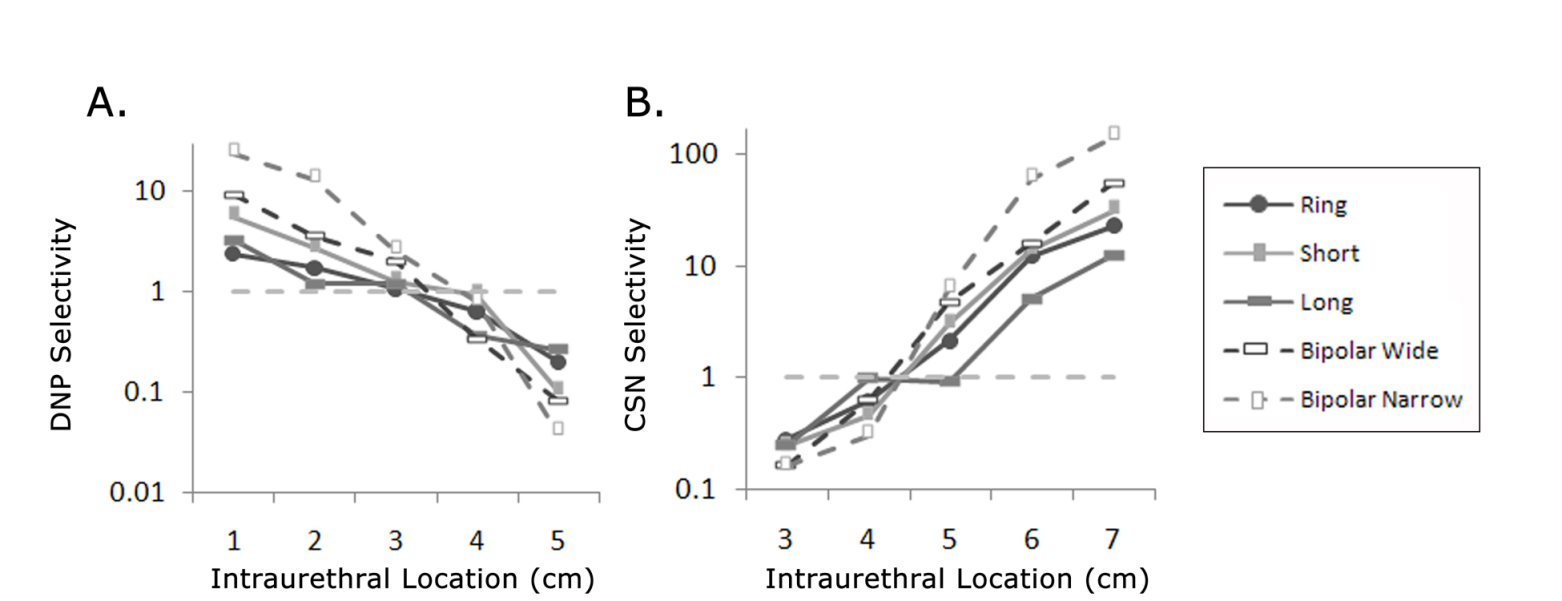
**Pudendal sensory branch selectivity**. (A) DNP selectivity (compared to CSN) as a function of electrode location for 1-5 cm from the urethral meatus. (C) CSN selectivity (compared to DNP) for 3-7 cm from the urethral meatus. (A, B) When selectivity was >1, AFRs were also >1.5.

The ability to activate the DNP selectively without co-activation of the CSN (and vice versa) was calculated for each electrode configuration at the different intraurethral locations (Figure 4). Selectivity for one nerve was greatest when the electrode was furthest from the other nerve (e.g., DNP selectivity was greatest at 1 cm from the urethral meatus, Figure 4A), and selectivity values for all electrode geometries were <1 at 4 cm for the DNP (Figure 4A) and CSN (Figure 4B). This reveals that at ~4 cm (the proximal portion of the penile bulb) neither the DNP nor the CSN can be activated selectively.

**Figure 5:**
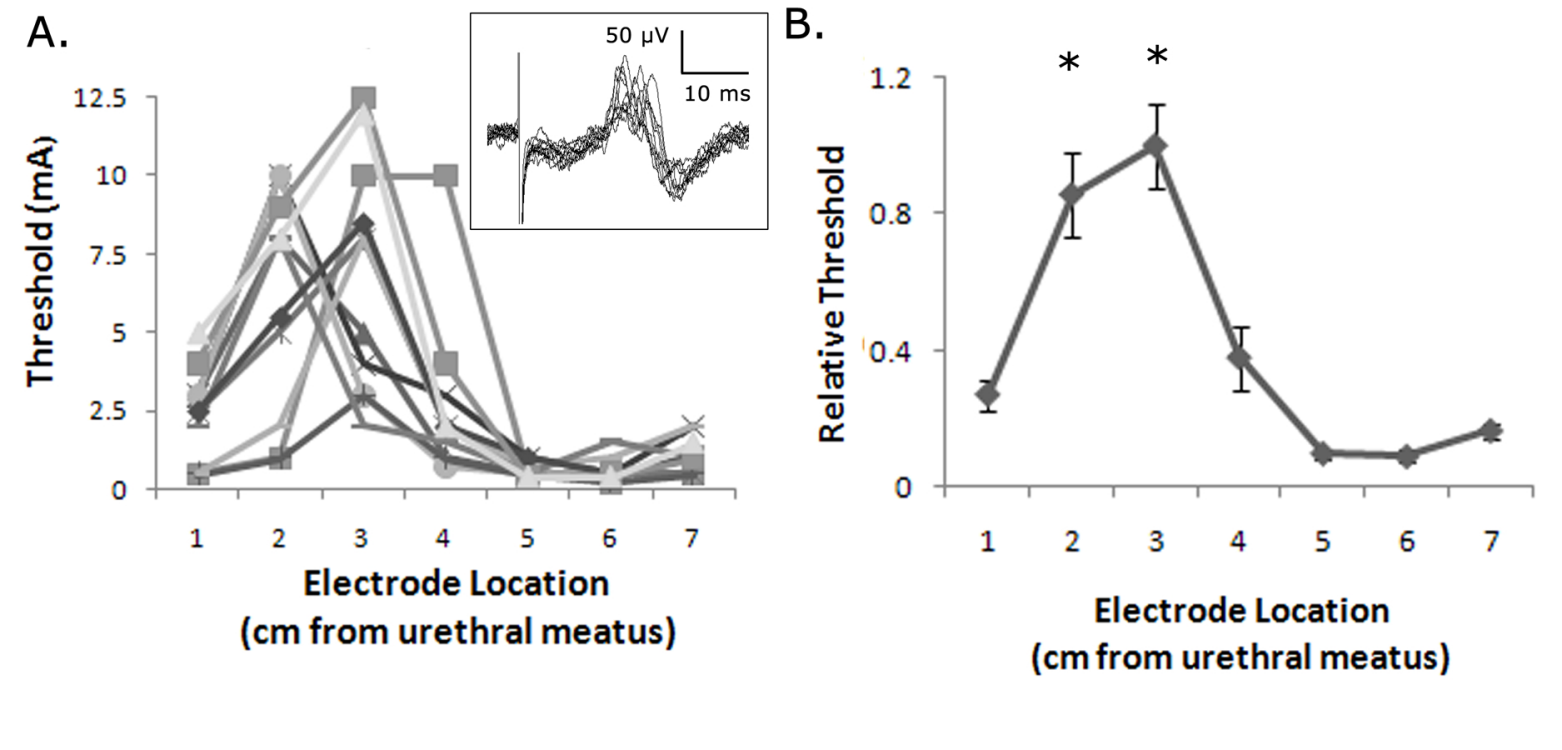
**In vivo thresholds to evoke reflex EAS activation by intraurethral stimulation**. (A) Stimulation thresholds for evoking an EAS reflex response in 11 cats with 1 mm or 2 mm ring electrodes. Inset: Reflex EAS EMG responses evoked by 1 Hz, 6 mA IES at 1 cm from the urethral meatus. (B) Normalized intensity thresholds were dependent on stimulation location (p < 0.001, one-way ANOVA), and stimulation at 2 and 3 cm required significantly higher thresholds to evoke an EAS reflex response than stimulation at all other locations (p < 0.05, Bonferroni post-hoc comparisons).

IES with a monopolar ring electrode (1 mm or 2 mm) evoked reflex EMG responses in the EAS in 11 of 11 cats. The threshold to evoke a response with a ring electrode was dependent on the intraurethral location (Figure 5, one-way ANOVA, p < 0.0001). The threshold intensity was significantly lower in the area of the glans penis (1 cm) and the membranous urethra (5-7 cm) than in the area of the penile body (2-3 cm). The average threshold intensity (Figure 5B) revealed a location-intensity relationship similar to that predicted by the finite element model (Figure 3D).

### Activation threshold depended on electrode geometry

**Figure 6:**
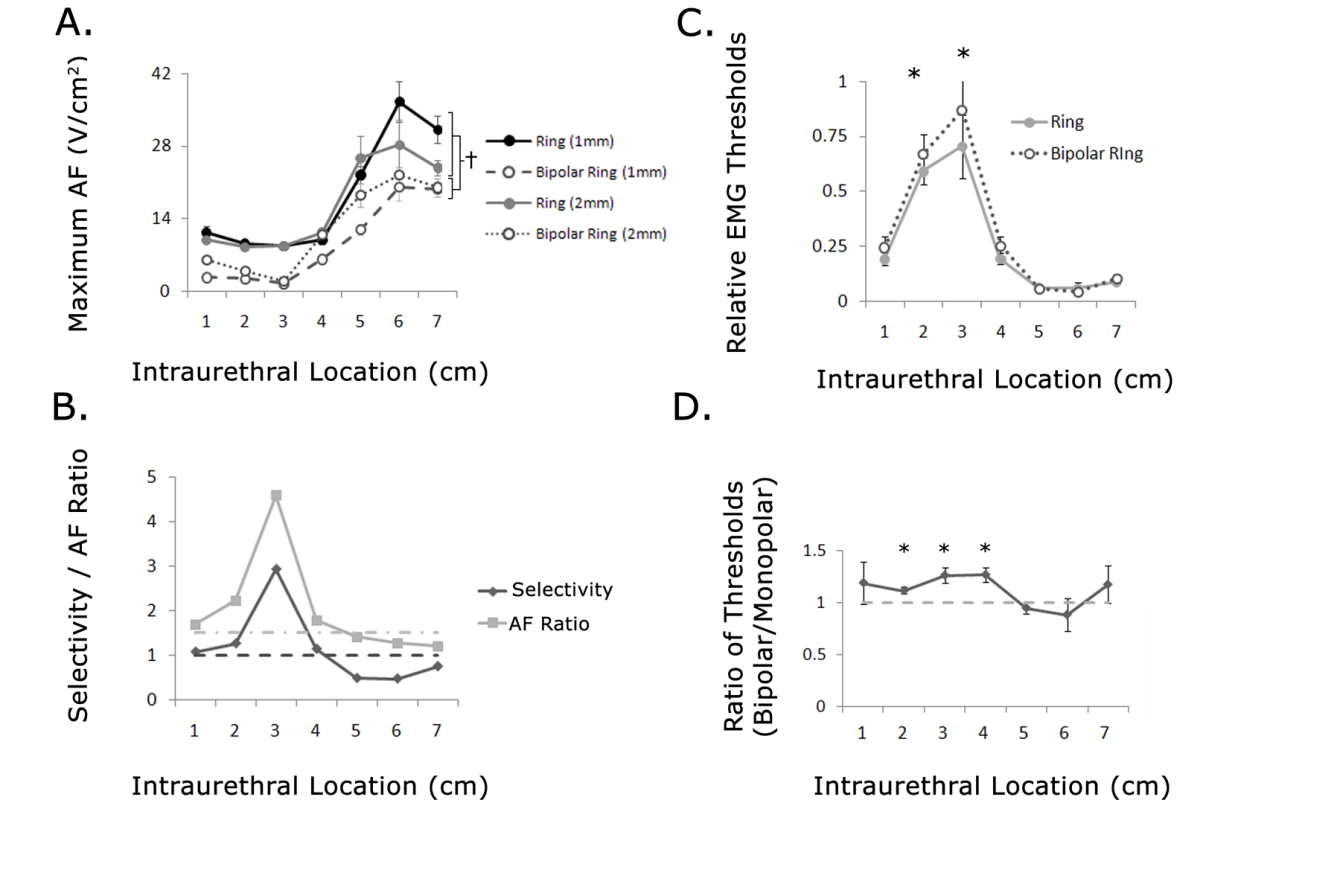
**Simulation and in vivo comparison of monopolar and bipolar ring electrodes**. (A) Maximum activating function (AF) along the DNP or CSN evoked by 1 mm and 2 mm monopolar and bipolar ring electrodes. The monopolar electrode configurations generated larger AFs than the bipolar configurations (†, p < 0.05, Bonferroni post-hoc comparison). (B) Selectivity and AF ratio for the 2 mm monopolar ring electrode compared to the 2 mm bipolar ring electrode. (C-D) In vivo stimulation intensity thresholds for evoking EAS EMG reflex responses with 2 mm monopolar ring and 2 mm bipolar ring electrode configurations. (C) Relative stimulation thresholds were dependent on stimulation location (p < 0.001, multi-way ANOVA) but not electrode configuration (p = 0.26). Stimulation at 2 and 3 cm required significantly higher thresholds to evoke an EAS reflex response than stimulation at all other locations (*p < 0.05, Bonferroni post-hoc comparison). (D) Averaged ratio of EAS threshold for bipolar and monopolar 2 mm ring electrodes at different stimulation locations. The ratio of EAS reflex thresholds (bipolar/monopolar) was significantly greater than 1 at 2, 3, and 4 cm from the urethral meatus (*p < 0.05, paired one-sided t-tests).

The maximum AFs were also dependent on the electrode configuration (two-way ANOVA, p < 0.0001). Intraurethral stimulation with the short monopolar and wide bipolar configurations generated larger AFs than stimulation with the other electrode configurations (Figure 3C, p < 0.05, post hoc comparisons with Bonferroni correction). Stimulation with 1 mm and 2 mm monopolar ring electrodes evoked significantly larger AFs than stimulation with 1 mm and 2 mm bipolar ring electrodes (Figure 6A, p < 0.05, post hoc comparisons with Bonferroni correction). Although the 1 mm monopolar electrode evoked larger AFs than the 2 mm monopolar electrode at all locations except 4 and 5 cm from the meatus, the differences were not significant (p > 0.05). Similarly, the 2 mm bipolar electrode evoked larger AFs than the 1 mm bipolar electrode at all locations, but the differences were also not significant (p > 0.05). Stimulation with the 1 mm and 2 mm bipolar ring electrodes evoked significantly smaller AFs than stimulation with all other electrode configurations (p < 0.05).

Comparison of the selectivity and AFR of the 2 mm monopolar and 2 mm bipolar ring electrodes revealed that bipolar stimulation required greater stimulation amplitudes to activate pudendal afferent fibers within the penile urethra (1-4 cm, Figure 6B), while no difference was predicted for stimulation in the proximal urethra. In vivo, the normalized stimulation thresholds for evoking an EAS response were not significantly different for bipolar ring electrodes and the monopolar ring electrodes (Figure 6C, two-way ANOVA, p = 0.17, n = 6 cats). However, the threshold ratios (bipolar electrode threshold divided by monopolar electrode threshold) revealed that thresholds were higher at 2-4 cm for the 2 mm bipolar ring electrode (Figure 6D), as predicted by the model simulations.

The modeled electrode geometries assumed that the electrodes were oriented in the direction of the nerve trunks and that electrode contact was flush with the urethral lumen. The effects of changing the orientation of the short electrode contact and modifying the diameter of the ring electrode were examined. Rotating the short contact electrode to face ventrally (away from the DNP) reduced the maximum AF by 30% for simulation of IES at 2 cm from the meatus. For the ring electrode, reducing the diameter of the stimulation catheter to one-half the diameter of the urethra (and filling the urethral cavity with urine, σ = 1.55) reduced the maximum AF by 20 and 30% for stimulation at 2 and 7 cm, respectively.

### DNP branch activation depended on electrode geometry

**Figure 7:**
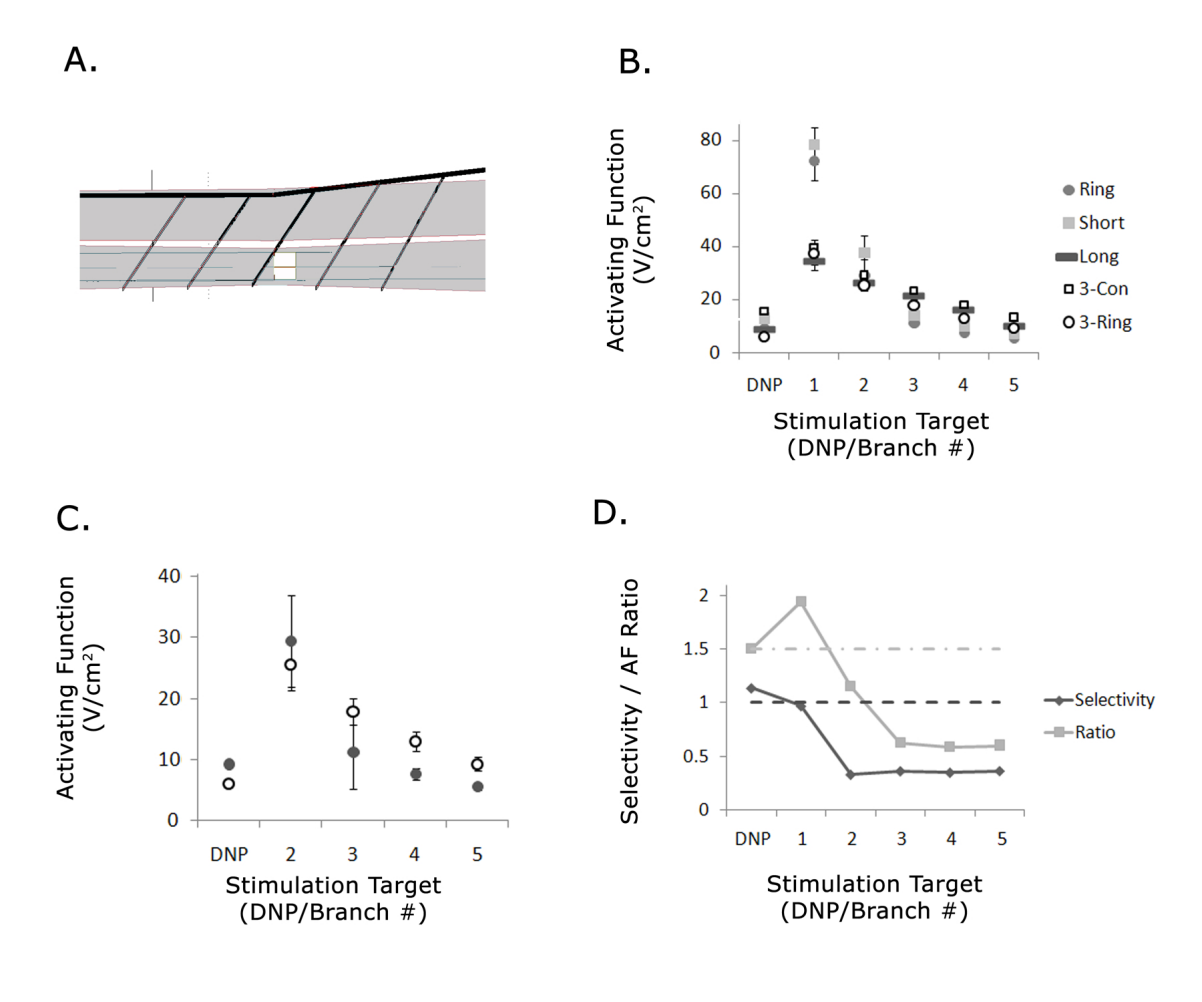
**Simulation of the effects of electrode geometry on activation of the trunk and lateral branches of the DNP**. (A) Lateral view of penile urethra from finite element model with simulated DNP branch locations (Figure 1). Each branch location was randomly varied between ± 1 cm along the urethra from the locations shown. (B) Maximum AFs evoked at the DNP trunk and in the DNP branches by stimulation at 2 cm from the urethral meatus. (C) Maximum AFs for the 1 mm ring and 3-ring electrodes only. The maximum branch AF ("Branch 1") was omitted for easier comparison of the AFs evoked by the different electrodes. (D) Selectivity and AF ratio for the ring electrode compare to the 3-ring electrode.

The DNP of the cat has lateral branches that project ventrolaterally along the penile body and innervate the urethra and perineum (Figure 7A) [19]. AFs generated by stimulation with 12 different electrode configurations (Figure 2A, B) positioned 2 cm from the urethral meatus were compared to determine the electrode configurations that could selectively activate these lateral DNP branches, without activating the DNP trunk and vice versa (Figure 7). Maximum branch AFs were averaged across trials based on their rank (i.e., the maximum AF across the 5 branches was averaged over the 50 trials and so on for the 2^nd ^largest, etc). AFs were dependent on the stimulation target (p < 0.0001, MANOVA). For all electrode configurations, the largest maximum AF generated over all the branches was greater than the maximum AF at the DNP trunk (Figure 7B, post hoc paired comparison by single-sided t-test, p < 0.001). However, the relative magnitudes of the 2^nd^-5^th ^maximum branch AFs varied compared to the maximum AF at the DNP trunk. For each electrode configuration, Table 2 shows how many branch AFs were greater than the DNP AF based on paired comparisons (single-sided t-test, p < 0.001) or selectivity and AFR (branch AF greater than DNP AF if selectivity > 1 and AFR > 1.5). The long, 5-contact, 3-ring, and 5-ring electrode configurations provided the most selective activation of the lateral DNP branches without activation of the DNP trunk, allowing for activation of ~4 branches, or ~0.9 cm of urethral length, while the ring electrode activated the fewest branches at lower thresholds than DNP activation. These results suggest that in vivo comparison of DNP stimulation thresholds with the ring electrode and the 3-ring electrode could provide evidence of whether activation of pudendal afferent fibers by IES in the penile urethra occurs at the lateral branches or at the DNP trunk (Figure 7C). The selectivity of the ring electrode compared to the 3-ring electrode (Figure 7D) indicated that the ring electrode stimulation threshold would be lower than the 3-ring electrode only in the case of DNP trunk activation.

**Table 2:** Number of lateral branches of the dorsal nerve of the penis (DNP) activated before DNP trunk activation as determined by statistical significance (*t*-test) or selectivity & activating function (AF) ratio

*Electrode Geometry*	p < .001	Selectivity>1, AF Ratio>1.5
Ring	2	1

Short	2	2

Long	4	4

Bipolar Narrow	3	2

Bipolar Wide	3	2

3-Contact	2	2

5-Contact	4	4

3-Ring	4	4

5-Ring	4	4

**Figure 8:**
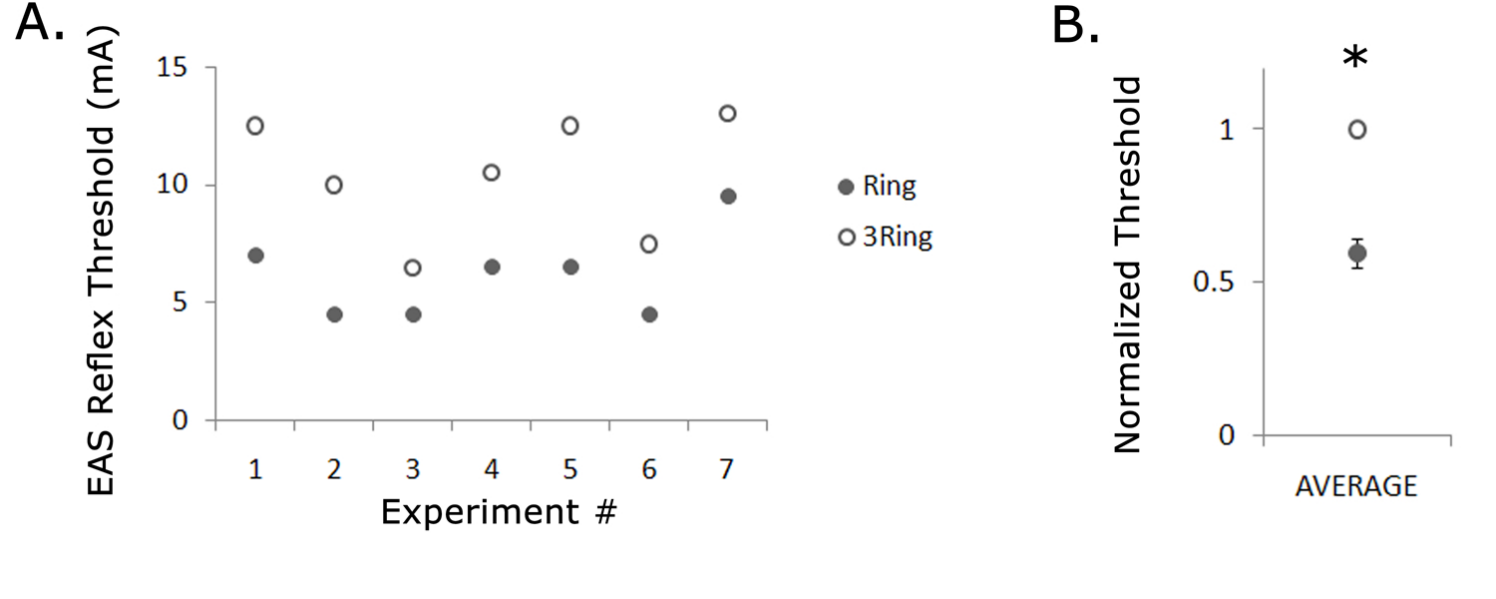
**In vivo intensity thresholds for ring and 3-ring electrodes at 2 cm from the urethral meatus**. (A) The thresholds for evoking an EAS reflex for stimulation at 2 cm from the urethral meatus were greater for stimulation with the 3-ring electrode than for stimulation with the ring electrode in 4 of 4 cats. (B) The normalized, average stimulation intensities revealed that stimulation with the ring electrode evoked an EAS reflex at significantly lower threshold than stimulation with the 3-ring electrode (*p < 0.0005, paired one-sided t-test).

In 7 of 7 cats, stimulation at 2 cm with the 1 mm ring electrode evoked a reflex EMG response in the EAS at a lower threshold than stimulation with the 3-ring electrode (Figure 8A). The normalized threshold for the ring electrode was 60% ± 5% of the normalized threshold for the 3-ring electrode (Figure 8B, p < 0.0005, paired one-sided t-test), and comparable to the simulation result that the ring electrode DNP trunk activation threshold is ~67% of the 3-ring electrode DNP trunk activation threshold. Thus, IES generates activation at the DNP trunk and not via the lateral branches along the length of the urethra.

## Discussion

Intraurethral electrical stimulation is a minimally invasive method to investigate the bladder responses evoked by activation of pudendal afferent fibers. However, the complex innervation of the urethra and surrounding structures makes it unclear what nerve branches are activated and how this varies with electrode geometry and location. The results of this study show that the location and geometry of the electrode both play significant roles in determining the stimulation threshold and selective activation of the two primary sensory branches (DNP, CSN) of the cat pudendal nerve. In this case, electrode location appears to be the primary factor in determining selectivity of activation. Also, the results indicate IES in the penile urethra activates the DNP trunk and not the lateral branches of the DNP.

This study provides a quantitative analysis of different electrode geometries for intraurethral stimulation, however there are several important limitations. First, the model is a simplified representation of the male feline lower urinary tract with neural innervation by the CSN and DNP. The nerves were represented as single trunks but the DNP has been shown to branch extensively in the area of the glans penis and the CSN typically has a lateral branch along the membranous urethra in addition to the medial branch modeled here [19]. Second, the AF is only an approximation of the relative thresholds for nerve activation by electrical stimulation [22]. Additionally, the fit between the urethra and the electrode contacts in vivo may vary, altering the current density at the different contacts in the multi-contact electrodes and ultimately affecting the thresholds for activation. However, the similarities between the in vivo stimulation thresholds and those predicted by the model demonstrate that the simplifications were justified to support our conclusions.

### Stimulation Location

Selective activation of the DNP was best achieved by stimulation in the distal urethra (near the glans) while selective activation of the CSN was best achieved by stimulation in the proximal urethra (near the prostate). Bladder responses evoked by intraurethral activation of pudendal afferent fibers also exhibit different characteristics for stimulation near the glans penis (high frequency [33-40 Hz] excitation; low frequency inhibition [5-10 Hz]) and near the prostate (excitation at all frequencies [2-33 Hz]) [15,16]. Further, these in vivo observations highlight the importance of selective DNP or CSN activation because the bladder response to activation of these nerves is different for different stimulation frequencies and involves different neural pathways [2].

The innervation of the urethra is spatially distinct [19,20]. IES can activate afferent fibers in the pudendal, pelvic, and hypogastric nerves, and the degree of activation of each nerve is dependent on intraurethral electrode location [20]. Innervation of the proximal urethra by autonomic nerve fibers from the pelvic and hypogastric nerves overlaps with the somatic innervation by CSN fibers [19,20], and IES in the proximal urethra may result in co-activation of pudendal and autonomic fibers. A previous study of intraurethral stimulation in the cat found that the pudendal and pelvic nerves were both activated by intraurethral stimulation in the membranous urethra [20]. The simulation and in vivo results show that the threshold for pudendal afferent fiber activation for stimulation in the proximal urethra (CSN activation) was lower than the threshold for stimulation in the penile urethra (DNP activation), so future clinical studies should investigate the use of lower amplitude stimuli in the proximal urethra compared to the penile urethra to avoid spillover of activation to neighboring nerves (e.g., autonomic innervation of the proximal urethra). The pudendal and pelvic afferent innervation of the urethra includes myelinated A-fibers and unmyelinated c-fibers. However, the myelinated pudendal urethral afferent fibers are larger, potentially consisting of Aα-, Aβ-, and Aδ-fibers, than the myelinated pelvic urethral afferent fibers, primarily Aδ-fibers [23,24]. These differences in fiber diameters suggest that it may be possible to limit co-activation of pelvic and hypogastric nerve afferent fibers by minimizing stimulation intensity, but the in vivo thresholds were sufficiently high to suggest co-activation may be difficult to avoid. Pelvic and hypogastric nerves were not modeled here, but should be considered in future work. The inability to distinguish pudendal and autonomic activation in the proximal urethra is of concern for clinical studies investigating the ability to evoke bladder responses via urethral pudendal afferent fiber activation. In a previous study, intraurethral stimulation evoked contractions in persons with spinal cord injuries [14], but effective stimulation locations were 2-4 cm from the bladder neck, and the roles of pudendal and autonomic nerve fibers in the observed response is unclear.

### Electrode Geometry

The different electrode geometries generated different AFs, which suggests that stimulation thresholds would be different for the different electrode geometries (Figure 3D). The short electrode configuration exhibited the lowest stimulation thresholds (determined by the comparing the inverse of the AFs), followed by the ring electrodes (1 and 2 mm), while the bipolar ring electrodes (1 and 2 mm) required higher stimulation intensities to activate the pudendal afferent fibers. The short electrode was directed dorsally, toward the nerve branches, and in practice the orientation of the electrode may be difficult to maintain. Further, the results confirm that improper orientation significantly increases stimulation threshold. Thresholds with the ring electrode would be more consistent although slightly higher than the thresholds for the ideally oriented short electrode. Previous studies of intraurethral activation in the cat found no difference between stimulation thresholds for monopolar and bipolar stimulation. However, one study focused on stimulation in the proximal urethra [14], which our results predicted would not have different thresholds, while the second study compared thresholds in different animals [16], which are unlikely to be significantly different because of interanimal variability. Further, contact size and spacing between contacts differentially affect the ability to activate pudendal afferent fibers (e.g., increasing contact length decreases AFs but increasing contact spacing increases AFs) so comparison of electrodes of varying lengths and spacing is complicated. In vivo thresholds were smaller for monopolar stimulation than bipolar stimulation, but the difference in threshold magnitude was less than that predicted by the model. Anatomical variability may confound this measurement in vivo because the location of the stimulation target with respect to the electrodes contributes to threshold differences between monopolar and bipolar stimulation [25].

The selectivity between activation of the DNP and the CSN was dependent on electrode geometry. The narrow bipolar electrode had the greatest selectivity, but selectivity values for all electrodes tended to be high near the urethral meatus and the prostate. No electrode geometry exhibited selectivity >1 at 4 cm from the urethral meatus, suggesting that stimulation in the penile bulb will produce co-activation of the CSN and DNP. In vivo investigations of the bladder response to intraurethral stimulation considered the effect of stimulation location but failed to address the potential for simultaneous excitation of the CSN and DNP [15,16]. Variability in the bladder response to intraurethral stimulation 4 cm from the urethral meatus led this distance to be excluded from quantification in our previous study of intraurethral stimulation in the cat [15], while 4-6 cm was grouped together in another study of IES [16]. Bladder responses evoked by IES in the penile and membranous urethra are abolished by bilateral transection of the DNP and CSN, respectively [15], indicating that IES at different intraurethral location allows minimally invasive selective activation of different pudendal afferent branches. A better understanding of IES will enhance our ability to target pudendal afferent branches selectively in clinical investigations. In addition to providing insight into the ability to restore control of bladder function, selective pudendal afferent activation via IES may be a useful tool for enhancing understanding of the physiology and pathophysiology of urinary dysfunction.

### Functional Significance of Lateral Branches of the DNP

A potential benefit of intraurethral stimulation verses transcutaneous (with external surface electrodes) or percutaneous stimulation of the DNP would be selective activation of urethral as opposed to cutaneous DNP fibers. Our results suggest that the range of activation of the lateral branches of the DNP without activation of the DNP trunk varies with electrode geometry. The lateral DNP branches are observed to give off branches that dive towards the urethra (sparsely) [19], and these urethral offshoots were not modeled, making our estimate of the impact of electrode configuration on urethral activation even more conservative. Based on these results, both clinical and experimental IES studies (which all utilize a ring electrode) are activating the DNP trunk, not the lateral branches. The 3-ring electrode could be further tested in vivo to determine if selective activation of the lateral DNP branches has any influence on the evoked bladder reflexes.

While intraurethral stimulation is an ideal means of activating urethral nerve fibers in the proximal urethra, percutaneous or transcutaneous stimulation of the DNP (or dorsal clitoral nerve) may be achievable at lower thresholds [26]. A previous experiment found that percutaneous, transcutaneous, and intraurethral stimulation (monopolar ring electrode) activated the DNP at 3-5 mA, 10-15 mA, and 15-25 mA, respectively [27]. Anatomical observations in humans and cats identified two populations of DNP axons [1,19,28], those travelling laterally on the penile body to the urethra and those travelling down the penile midline to the glans. If these populations play different roles in the inhibitory and excitatory bladder response to DNP stimulation, use of a 3-ring electrode configuration may be valuable for selective activation of the urethral fiber mediated reflex pathway. Transcutaneous DNP stimulation (with external surface electrodes) in humans evokes robust inhibition of the bladder [9-11], but selective activation of the urethral afferent fibers of the DNP may be necessary to evoke robust excitatory bladder responses in humans. This model relied on the detailed description of the innervation of the cat urethra. A thorough description of the innervation of the human urethra is needed to determine the ideal settings (electrode geometry and location) for clinical investigation of the bladder response to IES evoked selective activation of pudendal afferent branches.

## Conclusions

The threshold intensity to activate pudendal afferent fibers by IES is dependent on stimulation location and electrode configuration. Additionally, selective activation of the DNP or CSN depends on stimulation location. A ring electrode configuration is ideal for minimizing thresholds and variability in clinical and preclinical IES studies, but use of a multi-contact ring electrode provides a means of examining the specific role of distal urethral afferents in the bladder response to DNP stimulation.

## Competing interests

Warren Grill is an inventor on patents assigned to Case Western Reserve University and has consulting, grant support, and intellectual property relationships with Medtronic, Inc.

## Authors' contributions

All authors participated in the conception and design of the model and interpretation of data. JW acquired and analyzed the data and drafted the manuscript. All authors were involved in critical revision of the manuscript and have read and approved its final form.

## Pre-publication history

The pre-publication history for this paper can be accessed here:

http://www.biomedcentral.com/1471-2490/10/11/prepub
